# Cytokine activation is predictive of mortality in Zambian patients with AIDS-related diarrhoea

**DOI:** 10.1186/1471-2334-8-156

**Published:** 2008-11-13

**Authors:** Isaac Zulu, Ghaniah Hassan, Lungowe Njobvu RN, Winnie Dhaliwal, Sandie Sianongo, Paul Kelly

**Affiliations:** 1 Tropical Gastroenterology and Nutrition group, Department of Medicine, University of Zambia School of Medicine, Lusaka, Zambia; 2 Institute of Cell and Molecular Science, Barts & The London, School of Medicine, London, UK; 3 London School of Hygiene & Tropical Medicine, London, UK

## Abstract

**Background:**

Mortality in Zambian AIDS patients is high, especially in patients with diarrhoea, and there is still unacceptably high mortality in Zambian patients just starting anti-retroviral therapy. We set out to determine if high concentrations of serum cytokines correlate with mortality.

**Methods:**

Serum samples from 30 healthy controls (HIV seropositive and seronegative) and 50 patients with diarrhoea (20 of whom died within 6 weeks) were analysed. Concentrations of tumour necrosis factor receptor p55 (TNFR p55), macrophage migration inhibitory factor (MIF), interleukin (IL)-6, IL-12, interferon (IFN)-γ and C-reactive protein (CRP) were measured by ELISA, and correlated with mortality after 6 weeks follow-up.

**Results:**

Apart from IL-12, concentrations of all cytokines, TNFR p55 and CRP increased with worsening severity of disease, showing highly statistically significant trends. In a multivariable analysis high TNFR p55, IFN-γ, CRP and low CD4 count (CD4 count <100) were predictive of mortality. Although nutritional status (assessed by body mass index, BMI) was predictive in univariate analysis, it was not an independent predictor in multivariate analysis.

**Conclusion:**

High serum concentrations of TNFR p55, IFN-γ, CRP and low CD4 count correlated with disease severity and short-term mortality in HIV-infected Zambian adults with diarrhoea. These factors were better predictors of survival than BMI. Understanding the cause of TNFR p55, IFN-γ and CRP elevation may be useful in development of interventions to reduce mortality in AIDS patients with chronic diarrhoea in Africa.

## Background

The HIV and AIDS pandemic in sub-Saharan Africa is a major public health burden, causing high mortality and social disruption. In urban Zambian women aged 30–34 years, HIV seroprevalence is as high as 42.5% [1]. Recently, major progress has been achieved in rolling out highly active anti-retroviral therapy (HAART) in Zambia, but early mortality (within 30 or even 90 days of initiating HAART) remains a challenge [2]. The causes for this mortality are unknown, but may include undiagnosed opportunistic infections, nutritional impairment, or immune consequences of HIV which might not respond quickly to HAART. Even in developed countries, patients on HAART are still at increased risk of diarrhoea [3] and in much of sub-Saharan Africa, especially where HAART coverage is far from complete, diarrhoeal disease remains a major contributor to mortality.

We have previously shown that nutritional status is an important prognostic indicator in patients with AIDS-related diarrhoea[4], and there is a negative correlation between circulating soluble tumour necrosis factor receptors (TNFR) and nutritional status in these patients[5]. The driver of the cytokine activation remains uncertain but it seems likely that increased intestinal mucosal permeability in AIDS[6] allows translocation of bacteria or bacterial products into the portal vein, leading to Kupffer cell activation and release of IL-1β and TNFα[7]. Translocation into the lymphatic system or peritoneum can also occur[8]. Alternatively, HIV-driven macrophage and T cell activation may be responsible. Whatever the reason for cytokine activation, there is evidence that cytokine activation can predict outcome in AIDS patients in Europe and North America. In HAART-naïve Greek patients, sIL-2R in serum predicted time to death[9]. In a multivariate analysis of Spanish patients with advanced immunosuppression who had never received protease inhibitors, TNF-α independently predicted death[10]. As most previous work has been carried out in patients with less advanced disease, there is a poor understanding of predictors of outcome in patients with advanced immunosuppression, and it is in this group that most patients with diarrhoeal disease fall. Viral load does not predict outcome particularly well in this sub-group of patients[10]. We therefore decided to attempt to define cytokine predictors of mortality in African patients with diarrhoea. As previous work has tended to focus on a limited number of soluble markers, we attempted to encompass a wider range of molecules, including sTNFR (as a marker of TNF pathway activation), IL-6, IL-12, macrophage migration inhibitory factor (MIF), interferon-γ (IFN-γ) as well as C-reactive protein. We correlated concentrations of these cytokines (and the acute phase reactant) with 6-week mortality.

## Methods

### Study groups

Serum samples and data were drawn from two previous studies, one a trial of nitazoxanide in AIDS-related diarrhoea[11] and one a community study of small intestinal dysfunction from which control samples were obtained[6]. All the patients in the nitazoxanide clinical trial were HIV positive and had chronic diarrhoea. We therefore obtained HIV negative and positive asymptomatic controls from the community study of small intestinal dysfunction that had participants of a similar background to those in the nitazoxanide trial. These studies were approved by the Research Ethics Committee of the University of Zambia. They were carried out before the roll-out of the expanded access HAART programme over the last 2–3 years. In the nitazoxanide trial[11], no effect on mortality was seen. A total of 80 adults over 18 years of age, were divided into 5 groups (Table 1). Group 1 (n = 15) comprised healthy HIV seronegative adults drawn from a cohort study in Misisi compound, Lusaka[6]. None of these adults had evidence of infectious or digestive disease. Group 2 (n = 15) comprised HIV seropositive adults from the same cohort, with CD4 counts less than 200 cells/μl and without a history of any diarrhoea in the month prior to blood sampling. Groups 3–5 were drawn from a previously reported randomised controlled trial of nitazoxanide carried out in the University Teaching Hospital, Lusaka[11]. Group 3 (n = 15) comprised adult patients with HIV- related diarrhoea of more than one month duration and CD4 counts of 200 cells/μl or more. Group 4 (n = 15) comprised adults with HIV-related diarrhoea of over one month duration but CD4 counts under 200 cells/μl. All patients in groups 1–4 survived 6 weeks of follow up. Group 5 (n = 20) had HIV-related diarrhoea but died within 6 weeks of blood sampling.

**Table 1:** Characteristics of study groups

Group*	**HIV status**	**CD4 count (range)**	**Diarrhoea**	**BMI (kg/m^2^)**
1	negative	n/a	No	24.3 (sd 5.8)
2	positive	0–200	No	21.6 (sd 1.9)
3	positive	>200	Yes	16.6 (sd 2.0)
4	positive	0–200	Yes	16.5 (sd 2.5)
5	positive	0–200	Yes	16.2 (sd 2.9)

*Patients in groups 1–4 survived 6 weeks of follow up but patients in group 5 died during this period.

### Study procedures

After informed consent, patients were interviewed, had a physical examination, nutritional anthropometry and stool analysis performed for diarrhoea-causing intestinal pathogens as described elsewhere[6,11]. Pre and post test counselling for HIV was provided by a trained and experienced counsellor (LN). Blood was drawn for HIV testing and CD4 count, and aliquots of serum were stored at -80°C for cytokine and C- reactive protein (CRP) analysis. Blood draws from participants in both studies were conducted in the Gastroenterology clinic at the University Teaching Hospital in Lusaka; all participants were fasted and all blood samples were drawn between the hours of 0900 and 1200. Blood was collected into plain vacutainers, and refrigerated in the dark for between 1 and 3 hours until centrifugation.

All blood samples were treated the same way and collected by the same people using the same equipment and same protocol in the endoscopy unit because all these patients and even healthy controls were having endoscopies. After centrifugation, serum was separated and immediately stored at -80°C in the same ultra-low freezer until the assay was carried out. No freezer instability was experienced as Zambia has had (until now) an excellent electricity supply. This approach standardised the blood collection and processing procedure thereby reducing variation in serum cytokine levels influenced by method of collection and processing.

ELISA was used to quantify C-reactive protein (CRP; Kalon Biologicals, Aldershot, UK), Macrophage Migration Inhibitory Factor (MIF; Chemicon International, Temecula, Canada), Interferon-γ (IFN-γ), interleukin-12 (IL-12), interleukin-6 (IL-6) and soluble tumour necrosis factor receptor p55 (sTNFR; all from R&D systems, Abingdon, UK) according to the manufacturers' instructions. According to the manufacturers' datasheets the thresholds of detection of these assays were 0.2 mg/l, 1.6 ng/ml, 8.0 pg/ml, 5.0 pg/ml, 0.7 pg/ml and 0.77 pg/ml respectively.

### Statistical Analysis

Data were analysed using Stata version 8.2 (Stata Corp, College Station, Texas). Serum concentrations of CRP and the cytokines were not normally distributed, so analysis was performed using the Kruskal-Wallis test across all groups and Cuzick's non-parametric test for trend. While the primary analysis examined significance across all five groups, one post-hoc analysis was carried out and this was testing for significant differences between those who died and those who survived, using a Kruskal-Wallis test. We performed logistic regression to determine cytokines and their association with mortality with death at 6 weeks as a single endpoint. We dichotomised TNFRp55, cytokines and CRP around the median, body mass index (BMI) around 19 kg/m^2 ^and CD4 count around the value of 100 cells/ul. We included age and sex in the scale. To analyse further the effect of CD4 count we created categories of Cd4 count (<50, 50–100, 100–200, = 200 cells/μl) which could be used in logistic regression.

### Ethical considerations

The study was conducted in full accordance with the Declaration of Helsinki and informed consent was obtained. Approval for the studies was obtained from the University of Zambia Research Ethics Committee, and the study from which the control samples were drawn was also approved by the Research Ethics Committee of the London School of Hygiene and Tropical Medicine.

## Results

Table 1 describes the characteristics of the study participants. Serum samples from eighty patients (51 male, 29 female, mean age 29 years) were analysed and correlated with mortality at 6 weeks of follow-up.

### Serum cytokine and TNFR p55 concentrations at baseline

**Figure 1:**
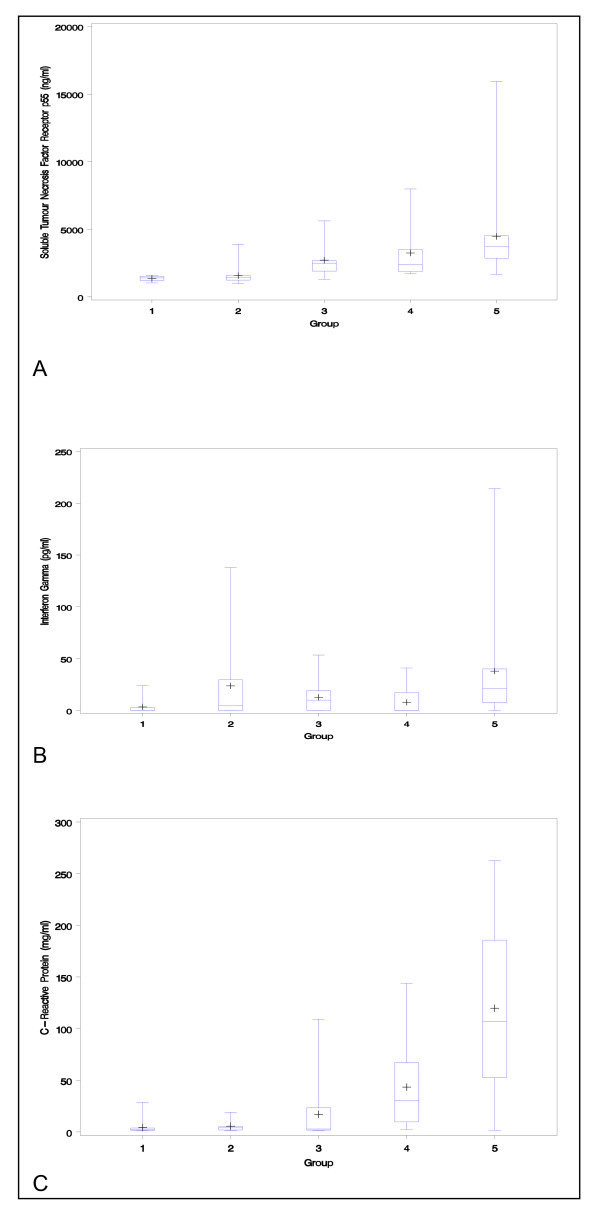
The box plots show median, interquartile range, and range of serum concentrations of sTNFR p55 (1A), IFN-γ (1B) and CRP (1C) in groups 1–5.

Serum cytokines and CRP were all readily detected in sera from the five groups, except for IL-12 which was below the threshold of detection of the assay in all samples tested and is therefore not discussed further. Concentrations of TNFRp55 (p < 0.0001), MIF (p < 0.005), IL-6 (p < 0.01), IFN-γ (p < 0.01), and CRP (p < 0.0001) all showed significant trends across all groups (Fig 1). Only TNFRp55 (p = 0.0001), interferon-γ (p = 0.0007) and CRP (p = 0.0001) were significantly greater in group 5 than in the other groups. This remained true if only patients with diarrhoea were considered: the significance or non-significance of each cytokine remained the same. As expected, BMI was lower in patients who died (16.2 kg/m^2^, SD 2.9) than in survivors (20.0 kg/m^2^, SD 4.9; p = 0.0006)

### Predictors of mortality at 6 weeks

Using logistic regression with death at 6 weeks as a single endpoint, and after dichotomising TNFRp55, cytokines and CRP around the median, these factors, together with body mass index <19 kg/m2, low CD4 count (CD4 count < 100 cell/ul), age and sex were analysed. In univariate analysis high sTNFR p55 (p = 0.001), IFN-γ (p = 0.001), CRP (p = 0.001), low CD4+ (p = 0.0001) and BMI (p = 0.018) were associated with mortality (Table 2). In multivariate analysis, adjusting for CD4 count, BMI and age, only sTNFR p55 (p = 0.033), IFN-γ (p = 0.013), CRP (p = 0.016) and low CD4 count (p = 0.01), remained independent predictors of mortality (Table 2). Further adjustment for CD4 count using categories as described in Methods confirmed the dependence of mortality on these biomarkers.

**Table 2:** Factors associated with mortality

	**Unadjusted OR (95% CI)**	**p**	**Adjusted OR 95% CI**	**p**
High sTNFR p55	31.90 (3.98–255.50)	0.001	10.4 (1.04–104.0)	0.05
High IFN-γ	6.0 (1.78–20.14)	0.001	8.14(1.5–43.7)	0.01
High MIF	2.26 (0.79–6.48)	0.12	0.53 (0.12–2.3)	0.4
High CRP	15.50 (3.29–73.4)	0.001	17.10 (1.71–171.0)	0.02
Low CD4+	13.52 (3.47–52.64)	0.0001	12.54 (1.75–89.62)	0.01
Low BMI	3.88 (1.25–12.03)	0.018	0.46(0.52–4.18)	0.50
Young age (< 40 years)	0.28 (0.67–1.17)	0.08	0.15(0.01–1.5)	0.11
Female	0.72 (0.19–2.64)	0.62	4.4 (0.39–49.2)	0.22

## Discussion

There is an urgent need to identify predictive factors for mortality in Zambian AIDS patients if we are to make any progress in reducing mortality attributable to HIV. While previous studies and ours have identified CD4 cell counts, opportunistic infections, sex, age, nutritional status and financial constraints as being associated with mortality in Africa[12,13], other factors such as immune activation have not been studied much in this region. A recent report from Zimbabwe shows that sTNF-rII independently predicted HIV disease progression and mortality[14]. Our data provide additional information indicating that cytokine activation may well make a major contribution to mortality in our patients. Of the five cytokines we studied, only IL-12 appeared not to be elevated in patients with AIDS related diarrhoea, but IL-6 and MIF were not directly related to mortality. The acute phase reactant CRP was also highly discriminating as a marker of disease severity and outcome. sTNFR p55 and interferon-γ showed appreciable and highly significant differences between groups of patients with AIDS related diarrhoea and different CD4 counts (groups 3 and 4).

In systemic sepsis patients in the critical care setting, IL-6 is probably the cytokine most closely related to outcome[15], but we did not find this. Unfortunately in that paper, interferon-γ was not measured, but they did observe that sTNFRp55 was a better predictor of outcome than TNFα itself, and in previous work we have found that sTNFRp55 correlates significantly with nutritional status[5]. It is of interest that in multivariate analysis, nutritional status was no longer predictive when cytokines were included in a regression model and it may be that in AIDS patients in Zambia, BMI merely reflects the effects of cytokine activation. There is also genetic evidence that TNF may play a causal role in HIV progression[16]. MIF concentration was elevated in patients with AIDS related diarrhoea, but was not independently related to mortality. MIF is constitutively expressed by a variety of cells and tissues, including monocytes and macrophages, and released rapidly on exposure to microbial products such as bacterial lipopolysaccharide (LPS) and TNF-α[17]. The intracellular pools of MIF are not only released more rapidly than TNF-α, but are signalled to do so at concentrations that are 10- to 100-fold lower than those required to induce TNF-α production. MIF has been detected in increased amounts in patients with severe sepsis and septic shock and has been demonstrated to be involved in pathogenesis of shock[18]. Inhibition of MIF protects experimental mice from lethal peritonitis[19]. It seemed of interest as a possible correlate of mortality, but we did not find this. The significance of CRP, which correlated extremely well with severity of disease and with mortality in our dataset, is probably that it behaves as a summation of inputs from several other cytokines, notably IL-1β, IL-6 and TNF. Although it is clearly a useful discriminator in analysing prognosis, it presents a very unlikely target for immunotherapy.

Therapy with cytokine modulators and antagonists is increasingly practised in many countries, beginning with anti-TNF antibodies for rheumatoid arthritis and Crohn's disease, and now for many other indications. In the realm of infectious disease, therapy directed at cytokine modulation has been less successful. Approaches using antibodies to lipopolysaccharide were disappointing[20]. The primary lesion which allows cytokine dysregulation has yet to be identified, though there are grounds for believing that it could begin with increased bacterial translocation across the intestinal mucosal barrier[7]. The high prevalence of blood-borne non-typhoidal salmonellosis in AIDS patients in Malawi lends further support to this contention[21]. The significance of cytokine activation for mortality which we describe here may suggest that it is appropriate to draw an analogy with the situation in critical care, where systemic sepsis is associated with cytokine over-activation.

Furthermore, cytokine activation may exacerbate the processes which gave rise to it. In this context, TNF and IL-6 can lead to increased T cell replication, presumably by up-regulation of molecules such as CCR5 on the surface of lymphocytes, thus facilitating viral entry. TNF also can cause enteropathy directly[22], which may accelerate the process of bacterial translocation. This is the rationale for using antibodies to cytokines and chemokines in systemic sepsis syndrome, and much work is ongoing in this area. Therapeutic modalities which reduce bacterial translocation may be able to arrest the cycle of cytokine derangements and gastrointestinal dysfunction in AIDS patients, and clinical trials of such therapies are urgently needed.

In the results section, the box plots show wide confidence intervals because a small number of patients showed extremely high values.

## Conclusion

High serum concentrations of TNFR p55, IFN-γ, CRP and low CD4 count correlated with disease severity and short-term mortality in HIV-infected Zambian adults with diarrhoea. These factors were better predictors of survival than BMI. Understanding the cause of TNFR p55, IFN-γ and CRP elevation may be useful in development of interventions to reduce mortality in AIDS patients with chronic diarrhoea in Africa.

## Competing interests

The authors declare that they have no competing interests.

## Authors' contributions

**IZ **was responsible for study design, data collection, cytokine assay, part of data analysis and writing of the manuscript

**PK **was responsible for study design, data collection, part of data analysis and writing of manuscript

**LN **was responsible for data collection, follow up of study patients and writing of manuscript

**WD **was jointly responsible for cytokine assays and writing of manuscript

**GH **was jointly responsible for cytokine assay and writing of manuscript

## Pre-publication history

The pre-publication history for this paper can be accessed here:


